# Zuranolone in Postpartum Depression: Mechanistic Innovation, Clinical Evidence, and Translational Uncertainty

**DOI:** 10.7759/cureus.114673

**Published:** 2026-08-17

**Authors:** Esteban Zavaleta-Monestel, Luis Guillermo Herrera-Jiménez, Katherine González-Aguilar, Jeaustin Mora-Jiménez, Ricardo Millán González

**Affiliations:** 1 Pharmacy, Hospital Clínica Bíblica, San José, CRI; 2 Pharmacy, University of Costa Rica, San José, CRI; 3 Research, Hospital Clínica Bíblica, San José, CRI; 4 School of Medicine, University of Costa Rica, San José, CRI

**Keywords:** antidepressant agents, gaba-a receptors, major depressive disorder, neuroactive steroids, perinatal mental health, pharmacotherapy, postpartum depression, zuranolone

## Abstract

Postpartum depression is a clinically significant perinatal mental health condition with consequences for maternal functioning, infant care, family dynamics, and healthcare engagement. Although psychotherapy, psychosocial support, and conventional antidepressants remain central to treatment, important therapeutic gaps persist, including the delayed onset of conventional antidepressants, variable or incomplete treatment response, tolerability concerns, and barriers to timely access to specialized perinatal mental healthcare, particularly when rapid symptom improvement is clinically needed. Zuranolone is an oral neuroactive steroid and positive allosteric modulator of gamma-aminobutyric acid type A receptors, administered as a short 14-day treatment course. Pivotal placebo-controlled trials demonstrated rapid short-term improvement in depressive symptoms among adults with postpartum depression, supporting its regulatory approval as an indication-specific treatment. However, the available evidence is strongest for short-term symptom reduction in trial populations with severe depressive symptoms, with the primary efficacy endpoint assessed at day 15 and follow-up extending only through day 45. Important uncertainties remain regarding durability of response, relapse, retreatment, breastfeeding outcomes, functional recovery, comparative effectiveness, access, cost, equity, and real-world implementation. In addition, zuranolone was not approved as a broad treatment for major depressive disorder, underscoring the need to avoid extrapolating postpartum depression findings to all depressive populations. Overall, zuranolone should be understood as a meaningful but evidence-bounded innovation in perinatal psychopharmacology, whose clinical value will depend on careful patient selection, safety counseling, follow-up, equitable access, and continued evidence generation.

## Introduction and background

Postpartum depression is a major depressive episode occurring after childbirth within the broader spectrum of perinatal depression. A meta-analysis of 565 studies across 80 countries and regions estimated a pooled global prevalence of 17.22%, although estimates varied substantially according to geographic region, income level, and assessment method [1]. Under the Diagnostic and Statistical Manual of Mental Disorders, Fifth Edition, Text Revision (DSM-5-TR), episodes beginning during pregnancy or within four weeks after delivery may receive the “with peripartum onset” specifier. Diagnosis requires at least five symptoms during the same two-week period, including depressed mood or loss of interest or pleasure, together with clinically significant distress or functional impairment [2].

Other features may include sleep or appetite disturbance, fatigue, psychomotor changes, impaired concentration, and feelings of guilt or worthlessness; symptoms should not be better explained by substances, another medical condition, or a manic or hypomanic episode. Because postpartum depression can disrupt maternal functioning, infant care, mother-infant bonding, family dynamics, and healthcare engagement, timely recognition and treatment are essential. Management may include psychotherapy, social support, and antidepressant pharmacotherapy, with treatment selection shaped by symptom severity, functional impairment, breastfeeding goals, patient preference, access to specialized care, and the need for timely improvement during a vulnerable caregiving period [2-4].

Because symptoms arise during a sensitive period for maternal-infant interaction, their clinical impact may be compounded by sleep disruption, limited social support, and socioeconomic adversity. These factors can increase illness burden and restrict access to timely diagnosis, specialized care, and newer treatments, particularly among patients with fewer financial and healthcare resources [3-6]. Against this background, pharmacological management of postpartum depression has traditionally relied on monoaminergic antidepressants, particularly selective serotonin reuptake inhibitors (SSRIs) and serotonin-norepinephrine reuptake inhibitors (SNRIs).

However, postpartum-specific randomized evidence remains limited and is concentrated mainly on SSRIs. A Cochrane review reported pooled SSRI response and remission rates of 55% and 42%, respectively, at five to 12 weeks, indicating that approximately 45% of participants did not meet the response criteria and 58% did not achieve remission; the certainty of these estimates was low or very low [7]. Therefore, conventional antidepressants remain important treatment options, but their effects are generally assessed over weeks rather than days, and a substantial proportion of patients experience incomplete response or non-remission. Moreover, these agents were not developed specifically for postpartum depression and act primarily through monoaminergic pathways, leaving a need for faster-acting, indication-specific, and mechanistically differentiated treatments. Brexanolone introduced such an approach as an intravenous neuroactive steroid, although its prolonged monitored infusion limits feasibility and scalability in routine postpartum care [8].

Zuranolone is an orally administered synthetic neuroactive steroid, a steroid-derived compound designed to modulate neuronal signaling, and acts as a positive allosteric modulator of gamma-aminobutyric acid type A (GABA-A) receptors. Preclinical studies indicate that it enhances GABA-mediated inhibitory currents across both synaptic and extrasynaptic GABA-A receptor subtypes, although the precise mechanism by which this modulation produces antidepressant effects in postpartum depression remains incompletely understood [9,10]. This pharmacological rationale is relevant because pregnancy and childbirth are accompanied by substantial neurosteroid fluctuations and adaptive changes in GABA-A receptor signaling that may contribute to postpartum vulnerability in susceptible individuals. However, these findings do not establish postpartum depression as a single-pathway neurosteroid disorder, given its heterogeneous and multifactorial pathophysiology [9,11].

The clinical development of zuranolone introduced a short-course oral neuroactive steroid option supported by evidence of rapid symptom improvement in adults with postpartum depression. In randomized placebo-controlled trials, zuranolone was associated with greater reductions in depressive symptom severity than placebo after a 14-day treatment course, supporting its regulatory approval for postpartum depression [12,13]. In August 2023, the U.S. Food and Drug Administration (FDA) approved zuranolone as the first oral medication indicated for the treatment of postpartum depression in adults [14].

The approved prescribing information recommends once-daily evening administration for 14 days and highlights important safety considerations, including central nervous system depressant effects and impaired ability to drive or engage in hazardous activities [9]. Nevertheless, approval does not resolve important therapeutic uncertainties regarding response durability, retreatment, management of partial response or relapse, sequencing or transition to conventional antidepressants, comparative effectiveness, breastfeeding safety, functional outcomes, real-world adherence, cost, coverage, equitable access, and optimal patient selection.

Zuranolone was also not approved as a broad treatment for major depressive disorder. The same regulatory decision included a Complete Response Letter for the major depressive disorder indication because the submitted evidence was not considered sufficient to support approval for that broader population [15]. This regulatory contrast argues against extrapolating findings from postpartum depression to depressive disorders more broadly. Therefore, zuranolone occupies a distinct position in contemporary neuropsychopharmacology as an indication-specific, mechanism-based innovation whose longer-term clinical role remains constrained by unresolved translational questions. This narrative review critically examines its neuropharmacological rationale, clinical evidence, safety profile, regulatory context, and translational uncertainties in postpartum depression.

## Review

Methodology

This article was designed as a critical narrative review, not as a systematic review or meta-analysis. The methodological approach was informed by the Scale for the Assessment of Narrative Review Articles (SANRA), which emphasizes a clearly justified topic, explicit objectives, transparent reporting of the literature search, appropriate referencing, scientific reasoning, and balanced presentation of relevant literature [16]. The review also followed principles of best-evidence synthesis for narrative reviews, prioritizing sources according to clinical relevance, methodological quality, and direct applicability to zuranolone and postpartum depression rather than pursuing exhaustive quantitative aggregation [17].

A structured literature search was conducted to identify sources relevant to zuranolone, postpartum depression, neuroactive steroid pharmacology, GABA-A receptor modulation, clinical efficacy, safety, regulatory status, lactation, and translational implementation. Searches were performed in PubMed/MEDLINE, with supplementary searches in Google Scholar and official regulatory or clinical sources, including the U.S. FDA, DailyMed, ClinicalTrials.gov, the American College of Obstetricians and Gynecologists (ACOG), the European Medicines Agency (EMA), and LactMed. Official regulatory and prescribing-information sources were prioritized for approval status, dosing, warnings, contraindications, controlled-substance status, lactation information, and labeling-specific safety statements. The literature search and review process was conducted from May through June 2026, with the final search completed on June 10, 2026. No formal publication date restriction was applied; the peer-reviewed and official sources included in the final review dated from 2006 to 2026. Only English-language publications and official documents were included, and no translated versions were used.

The search strategy combined free-text terms and, when applicable, controlled vocabulary related to postpartum depression and zuranolone. Search terms included “zuranolone,” “SAGE-217,” “postpartum depression,” “perinatal depression,” “neuroactive steroid,” “neurosteroid,” “GABA-A receptor,” “positive allosteric modulator,” “major depressive disorder,” “brexanolone,” “lactation,” “breastfeeding,” “safety,” “sedation,” “FDA approval,” and “complete response letter.” Terms were combined using Boolean operators according to the specific focus of each section of the review.

Eligible sources included randomized controlled trials, phase 2 and phase 3 studies, regulatory documents, prescribing information, clinical guidelines, systematic reviews and meta-analyses when useful for contextual interpretation, pharmacological reviews, mechanistic studies, and clinically relevant commentaries or debates. Particular emphasis was placed on primary clinical trial reports of zuranolone in postpartum depression, official prescribing information, FDA communications, and literature addressing neurosteroid biology, GABAergic signaling, and perinatal mental health.

Sources were excluded when they were not directly relevant to zuranolone, postpartum depression, neuroactive steroids, GABA-A receptor pharmacology, clinical safety, regulatory status, lactation, or real-world implementation. Non-peer-reviewed sources were not used for scientific claims unless they represented official regulatory documents, prescribing information, trial registry records, or manufacturer communications relevant to regulatory status. Duplicative publications, inaccessible records that could not be verified, and sources lacking sufficient bibliographic information were also excluded. Article selection and interpretation were reviewed collaboratively by the authors. Any uncertainty regarding the eligibility, relevance, or interpretation of a source was resolved through discussion and consensus among the author group; no formal third-party adjudication was required.

Evidence was synthesized thematically rather than quantitatively. The review was organized around predefined domains: clinical burden and therapeutic gaps in postpartum depression; mechanistic rationale for GABA-A receptor modulation; clinical evidence for zuranolone; safety and tolerability; lactation; regulatory contrast between postpartum depression and major depressive disorder; translational uncertainty; and clinical positioning. Within each domain, sources were interpreted according to clinical relevance and hierarchy of evidence. Randomized controlled trials and official regulatory sources were prioritized for efficacy and safety; clinical guidelines were used for therapeutic context; and mechanistic or pharmacological studies were used to support biological interpretation.

This methodology has limitations inherent to narrative reviews. A formal systematic review protocol was not registered, a formal risk-of-bias tool was not applied, and no meta-analysis was performed. The review should therefore be interpreted as a critical synthesis of available and clinically relevant literature rather than as an exhaustive systematic assessment. Nevertheless, the use of a structured search strategy, SANRA-informed reporting, explicit source prioritization, and reference verification was intended to improve transparency, traceability, and scientific rigor.

**Figure 1:**
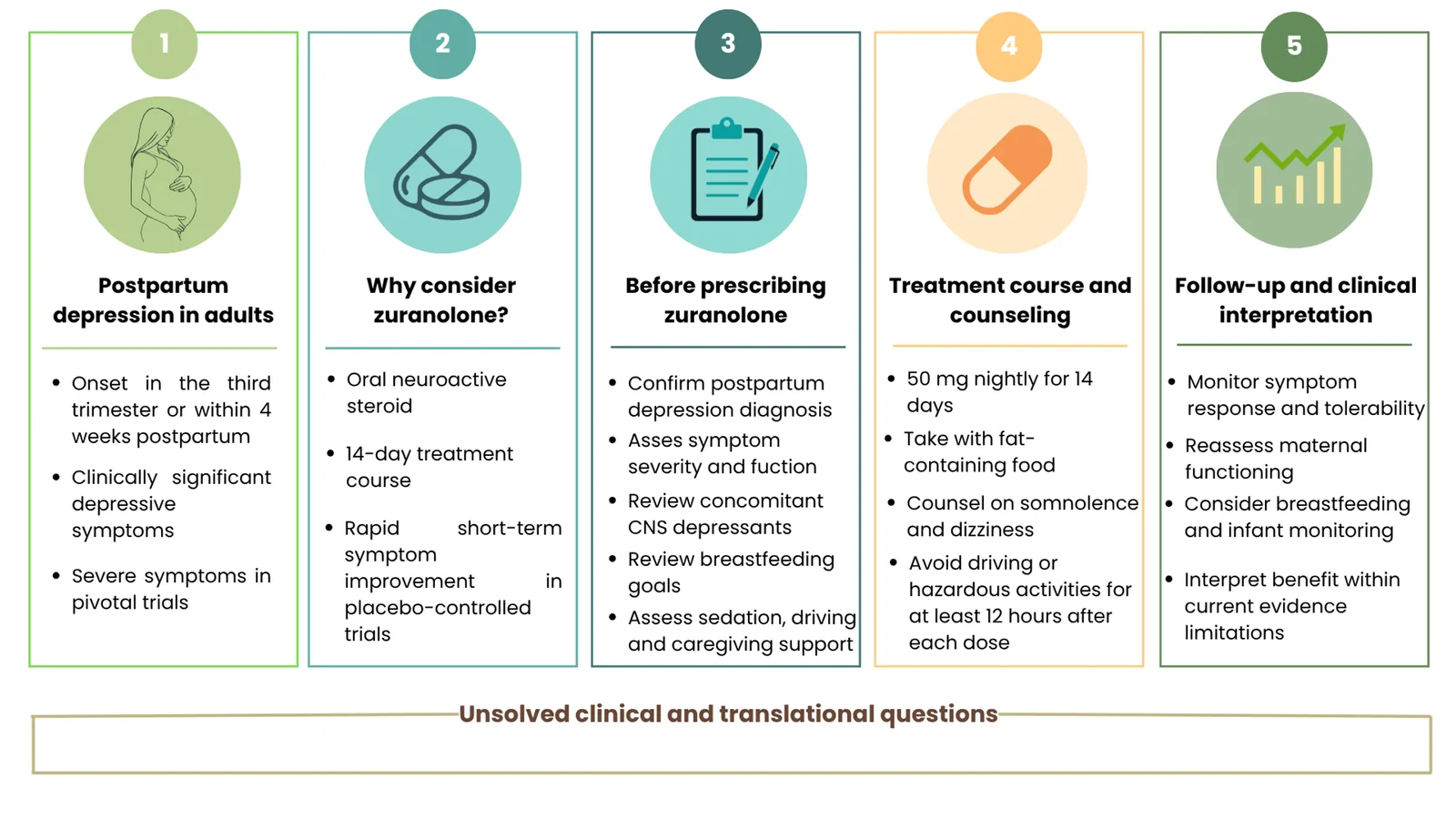
Clinical framework for interpreting zuranolone in postpartum depression. The framework presents five clinically relevant domains: identification of postpartum depression, the rationale for considering zuranolone, pre-prescribing assessment, treatment course counseling, and follow-up and clinical interpretation. The figure was created manually by the authors using Canva and standard non-generative graphic elements. No generative artificial intelligence tools were used to create or edit the figure. CNS: central nervous system

To guide the narrative synthesis, a clinically oriented framework for interpreting zuranolone across patient selection, treatment counseling, follow-up, and unresolved translational questions is presented in Figure 1.

Discussion

Clinical Burden and Current Therapeutic Landscape

Postpartum depression is a depressive disorder embedded in a distinctive biological, psychological, and caregiving context. Its clinical relevance extends beyond symptom severity, as maternal depressive symptoms during the postpartum period can interfere with functioning, mother-infant bonding, infant care, family dynamics, and engagement with healthcare services [3,4]. Contemporary clinical guidance emphasizes that treatment selection during pregnancy and the postpartum period should account for symptom severity, previous treatment response, patient preference, lactation, comorbid psychiatric symptoms, functional impairment, and access to care [4,18].

Before neuroactive steroid therapies became available, pharmacological management of postpartum depression relied primarily on conventional antidepressants, particularly SSRIs and SNRIs, often combined with psychotherapy and psychosocial support [4,18]. These treatments remain central to care but may not provide sufficiently rapid improvement when severe symptoms or marked functional impairment require an earlier clinical response. Brexanolone, an intravenous neuroactive steroid and positive allosteric modulator of GABA-A receptors, subsequently became the first mechanism-based treatment approved specifically for postpartum depression [4,8,18]. Brexanolone is chemically identical to endogenous allopregnanolone, a progesterone-derived neurosteroid involved in the regulation of inhibitory GABAergic signaling and known to fluctuate substantially across pregnancy and the postpartum transition [8,11,18]. However, its prolonged monitored infusion limited feasibility and scalability in routine care, and updated guidance from the ACOG notes that brexanolone is no longer commercially available in the United States [18].

Mechanistic Rationale: Neuroactive Steroid Modulation of GABA-A Signaling

Zuranolone differs pharmacologically from traditional monoaminergic antidepressants. It is a neuroactive steroid and positive allosteric modulator of GABA-A receptors [9,11,19]. This mechanism is clinically relevant because GABAergic inhibition contributes to neural excitability, stress responsivity, and affective regulation [9,11,19]. Experimental and translational studies have proposed that neurosteroid fluctuations during pregnancy and the postpartum period can contribute to vulnerability in susceptible individuals, particularly through changes in GABA-A receptor plasticity and inhibitory tone [9,11,19].

This biological rationale should not be read as evidence of a single causal pathway for postpartum depression. The disorder is clinically heterogeneous, and its pathophysiology is likely shaped by interactions among neuroendocrine, inflammatory, genetic, psychosocial, sleep-related, obstetric, and environmental factors [3,11]. Therefore, the mechanistic value of zuranolone lies not in explaining postpartum depression as a unified neurosteroid disorder, but in showing that GABA-A receptor modulation can produce measurable antidepressant effects in a defined postpartum population.

Clinical Evidence for Zuranolone in Postpartum Depression

The pivotal clinical data for zuranolone in postpartum depression come from two randomized, double-blind, placebo-controlled, multicenter trials involving adult women who met the DSM-5 criteria for a major depressive episode with symptom onset in the third trimester or within four weeks after delivery [9,12,13]. Both trials required baseline 17-item Hamilton Depression Rating Scale (HAMD-17) scores of at least 26, indicating that the studied populations primarily represented patients with severe depressive symptoms [12,13]. This distinction matters clinically because the FDA indication does not specify a severity threshold, whereas the trial populations were not designed to represent the full spectrum of postpartum depression severity [12,13,18]. The pivotal trials should also be interpreted as evidence from postpartum depression populations without bipolar disorder or active psychotic symptoms, because these higher-complexity presentations were excluded from the clinical development program [8,9]. This boundary is important in practice because postpartum depressive symptoms can occur in the context of bipolar-spectrum illness, and postpartum psychotic symptoms require urgent specialist assessment and a different therapeutic approach. Together, the two pivotal trials randomized 348 participants: 153 in the ROBIN study and 195 in the SKYLARK study.

In the ROBIN study, zuranolone was evaluated as a 14-day oral treatment course using an earlier investigational formulation. The trial randomized 153 patients, and the efficacy set included 150 patients. At day 15, zuranolone produced a greater reduction in HAMD-17 total score than placebo, with a least-squares mean change of −17.8 versus −13.6 and a between-group difference of −4.2 points (95% confidence interval (CI) = −6.9 to −1.5; p = 0.003) [12]. Differences favoring zuranolone were observed as early as day three and persisted through day 45 in the published trial report. Response and remission outcomes also favored zuranolone at day 15, although these secondary outcomes should be interpreted in light of the short treatment duration, limited sample size, and placebo-controlled design [12].

The SKYLARK study evaluated the 50-mg dose corresponding to the currently approved formulation. According to the prescribing information, Study 1 included 98 patients receiving 50 mg of Zurzuvae and 97 receiving placebo. Treatment was administered once daily in the evening for 14 days, with follow-up for a minimum of four weeks after treatment completion. At day 15, the least-squares mean change in HAMD-17 score was −15.6 with zuranolone and −11.6 with placebo, corresponding to a placebo-subtracted difference of −4.0 points (95% CI = −6.3 to −1.7). The published SKYLARK report similarly showed a statistically significant reduction in depressive symptom severity with zuranolone compared with placebo at day 15 [9,13].

The placebo-subtracted differences in both pivotal studies were statistically significant, but their clinical interpretation requires attention to context. A difference of approximately four points on the HAMD-17 may be clinically relevant for some patients, particularly when it occurs rapidly in a population with severe postpartum symptoms; however, rating-scale change should not be interpreted in isolation. A 2025 Cochrane review identified one zuranolone trial that assessed patient-reported maternal functioning using the Barkin Index of Maternal Functioning. At day 45, maternal functioning improved with zuranolone compared with placebo (mean difference = 7.20 points; 95% CI = 1.42-12.98; n = 153), although the certainty of this evidence was low, and no included study reported quality-of-life outcomes [20]. Evidence regarding sustained caregiving capacity, maternal-infant interaction, treatment satisfaction, and other patient-centered outcomes therefore remains limited. The pivotal trials support rapid short-term antidepressant efficacy, but the magnitude of benefit should be interpreted alongside response, remission, functional recovery, and the current limitations of patient-centered evidence [12,13].

Across the two pivotal trials, zuranolone demonstrated rapid improvement in depressive symptoms compared with placebo in adult patients with severe postpartum depression as represented in the clinical trial populations. The interpretation should remain short-term and indication-specific. The primary endpoint in both studies was assessed at day 15, and available follow-up was limited to approximately four weeks after completion of the 14-day treatment course [9,12,13,18]. The prescribing information states that safety and effectiveness beyond a single 14-day treatment course have not been established [9].

Thus, the available data support rapid short-term efficacy, but do not establish long-term durability, optimal management of relapse, repeated-course efficacy, or comparative effectiveness versus standard postpartum depression treatments. The available studies also do not define how clinicians should sequence or transition to conventional antidepressants or other therapeutic strategies when patients experience partial response, non-remission, symptom recurrence, or symptom worsening after completing the 14-day course.

Although the pivotal trials provide the main clinical foundation for zuranolone in postpartum depression, their findings should be interpreted within the limits of their design, population, comparator, and follow-up duration. Table 1 summarizes the key characteristics, efficacy findings, and limitations of these studies to support a balanced interpretation of the current evidence.

**Table 1:** Key clinical trials of zuranolone in postpartum depression. The ROBIN study evaluated zuranolone 30 mg, whereas the currently approved U.S. prescribing information recommends zuranolone 50 mg once daily in the evening for 14 days, with food containing fat [8,9]. DSM-5: Diagnostic and Statistical Manual of Mental Disorders, Fifth Edition; HAMD-17: 17-item Hamilton Depression Rating Scale; PPD: postpartum depression; SSRI: selective serotonin reuptake inhibitor; SNRI: serotonin-norepinephrine reuptake inhibitor

Study	Design	Population	Intervention	Comparator	Primary endpoint	Main findings	Key limitations
ROBIN; Deligiannidis et al., 2021; NCT02978326 [8]	Phase 3, double-blind, randomized, outpatient, placebo-controlled, multicenter trial conducted at 27 U.S. sites	Adult women aged 18-45 years, ≤6 months postpartum, with postpartum depression defined as a major depressive episode beginning in the third trimester or ≤4 weeks after delivery; baseline HAMD-17 score ≥26	Zuranolone 30 mg orally each evening for 14 days	Placebo	Change from baseline in HAMD-17 total score at day 15	The efficacy set included 150 patients. At day 15, zuranolone produced greater reduction in HAMD-17 total score than placebo: −17.8 vs −13.6; difference: −4.2 points; 95% CI: −6.9 to −1.5; p = 0.003. Differences favoring zuranolone were reported from day 3 through day 45	Short follow-up; placebo comparator only; severe symptom population; limited generalizability to milder postpartum depression; no active comparison with SSRIs/SNRIs, psychotherapy, or brexanolone; evaluated an earlier Zuranolone formulation/dose rather than the currently approved 50-mg regimen
SKYLARK; Deligiannidis et al., 2023; NCT04442503 [9]	Phase 3, randomized, double-blind, placebo-controlled, multicenter trial	Adult women with severe postpartum depression; DSM-5 major depressive episode with onset in the third trimester or within 4 weeks after delivery; baseline HAMD-17 score ≥26	Zuranolone 50 mg orally once daily in the evening for 14 days, with fat-containing food; dose reduction to 40 mg permitted for tolerability according to prescribing information	Placebo	Change from baseline in HAMD-17 total score at day 15	In the prescribing information efficacy analysis, 98 patients received zuranolone 50 mg and 97 received placebo. At day 15, least-squares mean HAMD-17 change was −15.6 with zuranolone and −11.6 with placebo; placebo-subtracted difference: −4.0 points; 95% CI: −6.3 to −1.7. The published trial reported significant improvement in depressive symptoms and general tolerability	Short follow-up; placebo comparator only; severe symptom population; limited evidence on durability beyond the acute follow-up period; no evidence establishing safety or effectiveness beyond a single 14-day treatment course; no direct comparison with standard antidepressants or psychotherapy

Anxiety, Insomnia, and Functional Outcomes

Exploratory analyses from the ROBIN study suggest that zuranolone may also improve concurrent anxiety and insomnia symptoms in women with postpartum depression [21]. These outcomes are clinically relevant because anxiety and sleep disturbance commonly coexist with postpartum depressive symptoms and may worsen functional impairment. However, these findings should be interpreted as exploratory rather than definitive. They raise the possibility that GABA-A receptor modulation may influence symptom domains beyond depressed mood, but they do not establish zuranolone as a primary treatment for postpartum anxiety or insomnia independent of postpartum depression [12,13,22].

Another clinically relevant gap concerns obsessive or intrusive symptom dimensions during the postpartum period. Intrusive or obsessional symptoms may occur in perinatal mood and anxiety presentations and can be highly distressing for patients, particularly when they affect maternal confidence, caregiving, or bonding. However, the available zuranolone trials and secondary analyses do not establish whether treatment specifically improves obsessive-compulsive symptoms or intrusive postpartum thoughts. Therefore, these symptoms should be actively assessed as part of routine clinical evaluation, but they should not be assumed to respond specifically to zuranolone based on the current evidence [12,13,22].

Patient-perceived functioning is another clinically important area, because postpartum recovery cannot be fully captured by rating scale changes alone. Improvements in depressive symptom scores may be meaningful only if they translate into better maternal functioning, caregiving capacity, sleep restoration, family participation, and engagement with ongoing care. Current trial data provide some supportive signals, but longer-term and real-world functional outcomes remain insufficiently characterized [12,13,18,21].

Safety, Tolerability, and Functional Considerations

The safety profile of zuranolone centers on central nervous system depressant effects. The prescribing information includes a boxed warning for impaired ability to drive or engage in potentially hazardous activities due to central nervous system (CNS) depressant effects. The label advises patients not to drive or perform activities requiring complete mental alertness until at least 12 hours after each dose during the treatment course, and notes that patients may be unable to judge their own degree of impairment. In postpartum care, this warning has practical implications beyond driving. Sleep deprivation, nighttime awakenings, infant handling, feeding, and the need to respond promptly to infant distress can increase the functional consequences of sedation, dizziness, slowed cognition, or impaired coordination. Safety counseling should therefore address driving restrictions, household support, and risk reduction during infant care throughout the 14-day course [9].

Because CNS depressant effects can be increased by concomitant alcohol, benzodiazepines, opioids, tricyclic antidepressants, or other sedating agents, medication reconciliation should be part of the pre-treatment assessment. This is particularly important in routine postpartum care, where exposure to sedating medications can occur despite efforts to avoid or minimize them. Before initiating zuranolone, clinicians should identify alcohol use, non-essential sedatives, opioid analgesics, hypnotics, anxiolytics, and other CNS depressants, and ensure that patients and caregivers understand the potential additive risks of sedation, impaired coordination, falls, reduced alertness, and compromised infant care safety [9].

In Study 1, somnolence occurred in 36% of patients receiving 50 mg of zuranolone compared with 6% receiving placebo. The most common adverse reactions reported more frequently with zuranolone than placebo were somnolence, dizziness, diarrhea, fatigue, nasopharyngitis, and urinary tract infection. Dose reduction due to adverse reactions occurred in 16% of patients receiving 50 mg in Study 1, most often because of somnolence or dizziness. Serious adverse reactions across postpartum depression clinical studies included confusional state in approximately 1% of patients [9].

Zuranolone is also classified as a Schedule IV controlled substance in the United States, and the prescribing information describes abuse potential and dependence-related concerns [9]. These considerations do not preclude appropriate use, but they support positioning zuranolone as a treatment that requires careful patient selection, counseling, medication review, and follow-up rather than as a low-complexity substitute for conventional antidepressant therapy.

Lactation and Reproductive Considerations

Pregnancy and lactation recommendations differ across regulatory and clinical sources. Clinical safety during pregnancy, including the third trimester, has not been established because available human exposure data are insufficient. The U.S. prescribing information warns that zuranolone may cause fetal harm based on animal studies, including neuronal injury after exposure during a developmental period corresponding to the human third trimester and early childhood, and recommends effective contraception during treatment and for one week after the final dose [9]. A phase 1 open-label study in healthy lactating individuals similarly reported low transfer of zuranolone into breast milk, with an estimated relative infant dose below 1% for the 50-mg dose [23]. LactMed concludes that low milk levels would not be expected to cause adverse effects in breastfed infants, but recommends careful infant monitoring until additional data are available, especially for newborn or preterm infants [24].

EMA similarly requires contraception during treatment and for seven days afterward but, unlike the U.S. label, formally contraindicates zuranolone during pregnancy [25]. Regarding lactation, both regulatory documents report low transfer into human milk, with a relative infant dose below 1%, but acknowledge limited or absent clinical outcome data in breastfed infants [9,23,25]. The U.S. label recommends an individualized assessment of the benefits of breastfeeding, the mother’s clinical need for treatment, and potential infant risks, whereas the EMA recommends discontinuing breastfeeding during treatment unless the healthcare professional determines that its benefits outweigh the possible risks to the child [9,25]. LactMed is comparatively more permissive, indicating that maternal treatment is not by itself a reason to discontinue breastfeeding, while recommending careful monitoring for excessive infant sedation, particularly in newborn and preterm infants [24].

These findings are reassuring from a pharmacokinetic perspective, but they do not establish clinical safety in a large cohort of breastfed infants. The available lactation study assessed drug transfer into milk rather than infant outcomes, and this distinction is clinically important. Lactation counseling should therefore be individualized rather than framed as a universal recommendation to continue or discontinue breastfeeding. In practice, shared decision-making may include either continuing breastfeeding during treatment with careful infant monitoring, particularly for excessive sedation, feeding difficulty, or reduced alertness, or temporarily pausing breastfeeding during the 14-day course and resuming after an appropriate post-treatment interval when this better aligns with maternal preference, infant vulnerability, local guidance, or risk tolerance [9,18,23,24].

This decision should account for maternal symptom severity, urgency of treatment, infant age and prematurity, breastfeeding goals, household support, and feasibility of monitoring. The central clinical question is not only how much zuranolone enters milk, but how best to balance maternal recovery, infant safety, and the psychological and relational value of breastfeeding in the postpartum period [9,18,23,24].

Regulatory Status and Indication-Specific Interpretation

Zuranolone was approved by the U.S. FDA in August 2023 as the first oral medication indicated for the treatment of postpartum depression in adults [14]. In 2025, the EMA reported that Zurzuvae received marketing authorization valid throughout the European Union for postpartum depression in adults following childbirth [25]. These regulatory decisions confirm the clinical and pharmacological significance of zuranolone as an indication-specific treatment for postpartum depression.

At the same time, regulatory interpretation must remain precise. Zuranolone was not approved by the FDA for major depressive disorder as a broad indication. Biogen and Sage Therapeutics reported that the FDA issued a Complete Response Letter for the major depressive disorder application, stating that the submitted evidence did not provide substantial evidence of effectiveness sufficient to support approval for that indication and that additional study or studies would be needed [15].

This regulatory contrast is central to the scientific interpretation of zuranolone. The drug may represent a meaningful innovation for postpartum depression without being established as a general antidepressant for all major depressive disorder populations. Regional regulatory documents may differ in specific implementation recommendations, particularly regarding lactation, reinforcing the importance of local prescribing guidance.

From a prescribing perspective, the recommended regimen is zuranolone 50 mg orally once daily in the evening with fat-containing food for 14 days; the dose may be reduced to 40 mg if CNS depressant effects occur. The U.S. label recommends 30 mg once daily in patients receiving strong cytochrome P450 3A4 inhibitors and in those with severe hepatic or moderate-to-severe renal impairment [9,25]. The U.S. prescribing information lists no formal contraindications, whereas the European product information contraindicates use during pregnancy and in patients with hypersensitivity to zuranolone or its excipients [9,25]. No routine laboratory monitoring is specified; however, medication reconciliation, assessment of renal and hepatic function when clinically relevant, and clinical monitoring for sedation, functional impairment, worsening depressive symptoms, and emergent safety concerns are appropriate. Detailed precautions and adverse effects are discussed in the preceding safety and reproductive considerations sections.

Real-World Implementation and Unresolved Uncertainties

The main translational uncertainties for Zuranolone concern durability, retreatment, comparative effectiveness, lactation, access, and implementation. The available data support symptom improvement after a single 14-day treatment course, but do not yet define longer-term outcomes [9,12,13,18]. ACOG notes that efficacy data beyond day 45 after treatment completion were not reported and that repeated treatment is not currently indicated when symptoms recur or fail to respond after a completed course [18]. This leaves an important clinical gap: patients may experience recurrence, partial response, or persistent functional impairment after the trial follow-up window, yet evidence-based strategies for retreatment or sequencing remain insufficiently defined.

Comparative effectiveness remains uncertain because the pivotal zuranolone trials used placebo rather than active comparators such as SSRIs, SNRIs, psychotherapy, brexanolone, or integrated perinatal mental healthcare [12,13,18]. A 2024 study used matching-adjusted indirect comparisons to compare the SKYLARK trial with randomized SSRI studies. In the Bucher indirect treatment comparison, zuranolone was associated with a 4.22-point greater reduction in Edinburgh Postnatal Depression Scale (EPDS) score than SSRIs at day 15 (95% CI = −6.16 to −2.28) and a 7.43-point greater reduction at day 45 (95% CI = −9.84 to −5.02); a network meta-analysis produced similar estimates [26]. These findings suggest a potential comparative advantage; however, they were not derived from head-to-head randomization, population overlap was limited, the effective sample size was substantially reduced after matching, and an indirect HAMD-17 comparison could not be performed. Accordingly, the analysis does not establish the superiority of zuranolone over SSRIs, and active comparator and pragmatic trials are still needed to define its relative role in clinical practice.

Access and cost will strongly shape the real-world impact of zuranolone. A clinical review in American Family Physician estimated that a 14-day course costs approximately US$19,000, substantially exceeding the cost of conventional generic antidepressants [27]. Equitable access in low- and middle-income countries will require more than regulatory authorization. Potential measures include context-sensitive pricing, transparent price negotiation, pooled or regional procurement, public reimbursement for appropriately selected patients, and health technology assessments that consider local budget impact and opportunity costs. These pharmaceutical policies should be accompanied by integration of perinatal mental health screening, diagnosis, and follow-up into maternal and child health services, training of non-specialist healthcare professionals, and clear referral and pharmacovigilance pathways. Until local affordability, comparative value, and implementation feasibility are established, access strategies for zuranolone should complement rather than displace scalable psychotherapy, psychosocial interventions, and established antidepressant treatments [28,29].

To clarify how these evidence gaps translate into clinical decision-making, Table 2 summarizes the main unresolved domains for zuranolone in postpartum depression, including durability of response, retreatment, comparative effectiveness, patient selection, lactation, functional recovery, access, equity, and pharmacovigilance.

**Table 2:** Main translational uncertainties for zuranolone in postpartum depression. CNS: central nervous system; EMA: European Medicines Agency; FDA: U.S. Food and Drug Administration; SNRI: serotonin-norepinephrine reuptake inhibitor; SSRI: selective serotonin reuptake inhibitor

Domain	Current evidence	Remaining uncertainty	Clinical implication
Durability of response	Pivotal studies demonstrated symptom improvement at the day 15 primary endpoint, with follow-up through approximately four weeks after the 14-day course [9,12,13]	Long-term durability beyond the available follow-up window remains insufficiently established	Patients should receive follow-up after treatment completion; symptom recurrence, incomplete response, or functional persistence of symptoms should be anticipated clinically
Retreatment and sequencing	The approved regimen is a single 14-day treatment course; prescribing information states that safety and effectiveness beyond one 14-day course have not been established [9]	Evidence is limited regarding repeated courses, retreatment timing, management of relapse, and transition to conventional antidepressant therapy after partial response, non-remission, or recurrence	Clinicians should not assume that repeated courses are evidence-based; sequencing with SSRIs, SNRIs, psychotherapy, or other therapeutic strategies should be individualized and monitored
Comparative effectiveness	Trials compared zuranolone with placebo [12,1 3]	Direct comparisons with SSRIs, SNRIs, psychotherapy, brexanolone, or integrated perinatal mental health care models are lacking	Zuranolone should not be framed as replacing established therapies; active-comparator and pragmatic studies are needed to define its role in routine care
Patient selection	Pivotal trials enrolled patients with severe symptoms, commonly baseline HAMD-17 ≥26, and excluded higher-complexity psychiatric presentations such as bipolar disorder and active psychotic symptoms [12,13,20]	Generalizability to mild or moderate postpartum depression, postpartum bipolar depression, psychotic presentations, broader comorbidity patterns, and diverse real-world populations remains uncertain	Careful diagnostic assessment, screening for bipolar-spectrum features, evaluation of psychotic symptoms, safety assessment, and specialist involvement are important before treatment selection
Symptom domains beyond depression	Exploratory analyses suggest possible improvement in concurrent anxiety and insomnia symptoms [22]	Evidence is insufficient regarding specific effects on intrusive, obsessional, or obsessive-compulsive symptom dimensions in the postpartum period [22]	Anxiety, insomnia, intrusive symptoms, and obsessive-compulsive symptoms should be assessed clinically, but zuranolone should not be assumed to treat these domains independently of postpartum depression
Functional recovery and maternal-infant outcomes	Symptom scales improved in pivotal trials [12,13]. Postpartum depression may affect maternal functioning, bonding, caregiving capacity, and infant-related outcomes [1-3]	Less is known about sustained maternal functioning, caregiving capacity, maternal-infant bonding, sleep restoration, quality of life, treatment satisfaction, and infant-related outcomes after zuranolone	Future studies should include patient-centered, family-centered, and dyadic outcomes, not only depression rating scales
Safety and postpartum functioning	Labeling emphasizes CNS depressant effects, somnolence, dizziness, confusional state, impaired coordination, and impaired ability to drive or engage in hazardous activities [9]	Real-world safety in sleep-deprived postpartum patients with variable household support, nighttime caregiving demands, and possible exposure to other CNS depressants remains incompletely characterized	Counseling, driving precautions, review of CNS depressants, household support planning, and risk reduction during infant care are essential
Lactation	Prescribing information and a phase 1 lactation study indicate low zuranolone transfer into human milk; LactMed also reports low milk levels [9,23,24]	Clinical outcomes in breastfed infants, especially newborns or preterm infants, remain limited. The emotional and relational impact of temporarily interrupting breastfeeding may also vary between patients [30]	Lactation counseling should use shared decision-making. Options may include continuation with careful infant monitoring or temporary interruption when clinically preferred, considering maternal benefit, infant vulnerability, breastfeeding goals, local guidance, and monitoring capacity
Regulatory scope	FDA approved zuranolone for postpartum depression in adults; EMA also lists Zurzuvae for postpartum depression in adults [14,25]	FDA did not approve zuranolone for major depressive disorder as a broader indication [15]	Findings from postpartum depression should not be extrapolated automatically to all major depressive disorder populations
Access, cost, and equity	Oral administration may reduce logistical barriers compared with monitored intravenous infusion [8,18]. A clinical review estimated the cost of a 14-day course at approximately US$19,000 [30]	Coverage, affordability, clinician familiarity, diagnostic access, and availability across health systems remain uncertain	Real-world impact will depend on implementation pathways, reimbursement, specialty-pharmacy access, post-treatment monitoring, and equitable access
Pharmacovigilance	Clinical trials and labeling identify key short-term safety issues [9,12,13]	Less is known about rare events, adherence patterns, misuse risk, concomitant medication use, and outcomes in broader populations	Post-marketing surveillance and real-world registries will be important for defining longer-term safety and use patterns

Table 3 translates the current evidence into practical considerations for patient selection, treatment, safety counseling, lactation, follow-up, and implementation, while positioning zuranolone as an indication-specific option rather than a replacement for established postpartum depression treatments.

**Table 3:** Evidence-to-practice summary of zuranolone in postpartum depression. CNS: central nervous system; GABA-A: gamma-aminobutyric acid type A; MDD: major depressive disorder; PPD: postpartum depression; SNRI: serotonin-norepinephrine reuptake inhibitor; SSRI: selective serotonin reuptake inhibitor

Domain	Evidence-supported interpretation	Clinical implication
Approved indication	Zuranolone is approved for postpartum depression in adults, but not as a broad treatment for major depressive disorder [6,10,11]	Findings should be interpreted as indication-specific and should not be extrapolated automatically to all depressive disorders
Mechanism	Zuranolone is an oral neuroactive steroid and positive allosteric modulator of GABA-A receptors [6,7,15]	It represents a mechanistically differentiated approach from conventional monoaminergic antidepressants, but mechanism alone should not guide treatment selection
Patient selection	Pivotal trials primarily enrolled adults with severe postpartum depression and excluded higher-complexity psychiatric presentations such as bipolar disorder and active psychotic symptoms [8,9]	Careful diagnostic assessment is essential, including evaluation of bipolar-spectrum features, psychotic symptoms, safety concerns, breastfeeding goals, prior treatment response, household support, and feasibility of follow-up
Treatment course	The approved regimen is a 14-day oral course, taken once daily in the evening with fat-containing food [6]	Counseling should emphasize timing, adherence, sedation risk, avoidance of CNS depressants when possible, and practical support during the treatment period
Clinical efficacy	Pivotal placebo-controlled trials showed rapid short-term improvement in depressive symptoms, with the primary endpoint assessed at day 15 [6,8,9]	The evidence supports short-term symptom reduction, particularly in trial populations with severe depressive symptoms; clinical significance should be interpreted alongside remission, functioning, maternal-infant bonding, and patient-centered outcomes
Durability and retreatment	Safety and effectiveness beyond a single 14-day course have not been established [6]	Long-term response, relapse management, repeated-course use, and transition to conventional antidepressant therapy when needed remain important evidence gaps
Safety and postpartum functioning	Central nervous system depressant effects, including somnolence, dizziness, impaired coordination, and impaired ability to drive or perform hazardous activities, are clinically relevant [6]	Patient counseling, driving precautions, review of CNS depressants, household support planning, and risk reduction during infant care are essential
Lactation	Available data suggest low transfer into human milk, but infant outcome data remain limited [6,18,19]	Breastfeeding decisions should be individualized. Options may include continuation with careful infant monitoring or temporary interruption when clinically preferred, with counseling that considers maternal benefit, infant vulnerability, breastfeeding goals, and local guidance
Comparative effectiveness	Pivotal studies compared zuranolone with placebo rather than SSRIs, SNRIs, psychotherapy, brexanolone, or integrated care models [8,9,14]	Zuranolone should not be framed as replacing established therapies; active-comparator and pragmatic studies are needed to define its role relative to SSRIs, SNRIs, psychotherapy, and integrated perinatal mental health care
Real-world implementation	Access, cost, coverage, clinician familiarity, follow-up capacity, and equity remain unresolved implementation issues. A clinical review in American Family Physician estimated the cost of a 14-day course at approximately US$19,000, substantially exceeding the cost of conventional generic antidepressants [1,2,14,21]	Its population-level impact will depend on health-system readiness, appropriate patient selection, reimbursement pathways, specialty-pharmacy access, household support, and post-treatment monitoring

Clinical Interpretation and Research Priorities

Taken together, the available evidence positions zuranolone as a meaningful but indication-specific innovation in postpartum depression. Its clinical value lies in combining a distinct neuroactive steroid mechanism, oral administration, and a short treatment course with evidence of rapid symptom improvement in adults with severe postpartum depressive symptoms. However, this promise should remain evidence-bound. Zuranolone should not be interpreted as a universal antidepressant breakthrough, nor should its postpartum depression evidence be extrapolated automatically to broader depressive populations.

Clinically, the central tension is between rapid symptom reduction and sustained postpartum recovery. The available trials support short-term improvement, but they do not establish durable remission, relapse prevention, repeated-course efficacy, long-term functional recovery, or superiority over established treatments. Postpartum recovery should therefore be assessed beyond depression rating scales, including maternal functioning, caregiving capacity, sleep, safety, treatment satisfaction, maternal-infant bonding, quality of life, lactation experience, and infant-related outcomes [1-3,6,8,9,14].

The next phase of research should move beyond short-term placebo-controlled efficacy and determine how zuranolone can be integrated into routine postpartum depression care. Active comparator trials are needed to compare zuranolone directly with SSRIs, SNRIs, psychotherapy, and combined-care strategies [6,8,9,14]. Pragmatic trials should evaluate effectiveness, adherence, safety, and feasibility under usual-care conditions, while prospective longitudinal cohorts and post-marketing registries should examine durability of response, relapse, retreatment, rare adverse events, concomitant medication use, and longer-term maternal and infant outcomes. Dedicated lactation studies should include breastfed-infant outcomes rather than milk concentrations alone. Implementation and cost-effectiveness studies are also needed to assess affordability, reimbursement, health-system readiness, and equitable access across different income settings.

Strenghts and Limitations

This review has several strengths. Its SANRA-informed and clinically oriented approach integrates evidence from pivotal randomized controlled trials, regulatory documents, prescribing information, clinical guidance, pharmacological studies, and lactation sources. Priority was given to primary trials and official sources for efficacy, safety, dosing, and regulatory claims, while the synthesis considered not only symptom reduction but also functional recovery, lactation, patient selection, access, equity, and real-world implementation. The clinical framework and evidence-to-practice tables further translate the available evidence into practical considerations while clearly distinguishing established findings from unresolved questions.

This review has limitations inherent to its narrative design. Although the search strategy was structured and informed by SANRA and best-evidence synthesis principles, the review was not conducted as a systematic review, did not include a registered protocol, did not apply a formal risk-of-bias tool, and did not perform a meta-analysis [12,13]. Study selection prioritized clinical relevance, hierarchy of evidence, and direct applicability to zuranolone, postpartum depression, neuroactive steroid pharmacology, safety, regulation, lactation, and implementation. The synthesis should therefore be read as a critical, clinically oriented appraisal rather than an exhaustive quantitative assessment.

The conclusions are also constrained by the available literature. Pivotal studies provide short-term placebo-controlled efficacy data, while real-world evidence, long-term durability, retreatment strategies, comparative effectiveness, breastfeeding outcomes, patient-centered recovery, dyadic outcomes, and implementation data are still insufficient to define the full clinical role of zuranolone [6,8,9,14,18,19]. These limitations do not negate the importance of zuranolone as an indication-specific pharmacological advance, but they define the boundaries within which its clinical use should be interpreted.

## Conclusions

Zuranolone represents a meaningful advance in the pharmacological treatment of postpartum depression by introducing an oral, short-course, mechanism-based therapy centered on neuroactive steroid modulation of GABA-A receptor signaling. Its approval marks an important shift in perinatal psychopharmacology and offers a rapid treatment option for a clinically vulnerable population. However, its interpretation should remain evidence-bound, as current data primarily support short-term efficacy in trial populations with severe depressive symptoms, while uncertainties remain regarding durability, relapse, retreatment, breastfeeding outcomes, comparative effectiveness, functional recovery, access, cost, and real-world implementation. Therefore, zuranolone should be understood as an indication-specific innovation rather than a universal antidepressant breakthrough. Its clinical value will depend on careful patient selection, safety counseling, follow-up, and equitable access. In low- and middle-income countries, applicability may be constrained by acquisition costs, limited reimbursement, specialist availability, and the capacity for diagnosis and post-treatment monitoring; access strategies should therefore complement rather than displace scalable established treatments. Future research should include head-to-head and pragmatic randomized controlled trials, prospective longitudinal cohorts, long-term post-marketing registries, dedicated lactation studies with infant outcomes, and implementation and cost-effectiveness studies. These designs are needed to determine whether rapid symptom improvement translates into sustained, functional, and patient-centered recovery across diverse healthcare settings.
